# Evolving Redo Frame-Preserving Leaflet Resection: From Transcatheter to Surgical Aortic Valves

**DOI:** 10.1016/j.atssr.2025.11.028

**Published:** 2025-12-19

**Authors:** Brian J. Solomon, Andreas Sarantopoulos, Travis Howard, Robert J. Cubeddu, Thomas Caranasos

**Affiliations:** 1 Department of Cardiothoracic Surgery, NCH Heart Institute, NCH Healthcare System, Naples, Florida; 2 Division of Cardiology, Rooney Heart Institute, NCH Healthcare System, Naples, Florida

## Abstract

**Background:**

Redo aortic valve replacement after bioprostheses failure carries high risk from friable tissue, pannus ingrowth, and potential root reconstruction. Building on the Surgical Resection of Prosthetic Valve Leaflets Under Direct Vision (SURPLUS) technique for failed transcatheter valves, we describe a novel evolution for failed surgical bioprostheses: Surgical Valve Preservation and Resection of Bioprosthetic Leaflets (SUPERB). In SUPERB, degenerated leaflets are excised while the supporting ring is preserved, permitting deployment of a balloon-expandable prosthesis under direct vision.

**Methods:**

Between January 2023 and August 2025, 3 high-risk patients with degenerated aortic valves underwent redo replacement at a tertiary center: 2 with failed surgical bioprostheses (SUPERB) and 1 with a failed transcatheter prosthesis (SURPLUS). All were unsuitable for valve-in-valve transcatheter replacement due to low coronary heights, pannus ingrowth, or small annuli. Operative details, perioperative course, and follow-up echocardiography were reviewed.

**Results:**

All patients underwent successful leaflet excision with balloon-expandable valve deployment and confirmed coronary patency. Cross-clamp and cardiopulmonary bypass times were minimized vs conventional explantation, and no annular disruption or root replacement was required. Postoperative complications included transient acute kidney injury (n = 2), delirium (n = 1), hypotension (n = 1), and urinary retention (n = 1). All were discharged to rehabilitation. At follow-up, the prostheses remained functional with preserved ventricular performance and no valvular regurgitation.

**Conclusions:**

The SUPERB technique extends the SURPLUS concept to failed surgical bioprostheses, providing hybrid alternatives to complete prosthesis explantation. These strategies may reduce operative risk by shortening cross-clamp and bypass times, preserving coronary access, and avoiding root reconstruction. Multicenter evaluation is warranted.

## Visual Abstract

**Figure:**
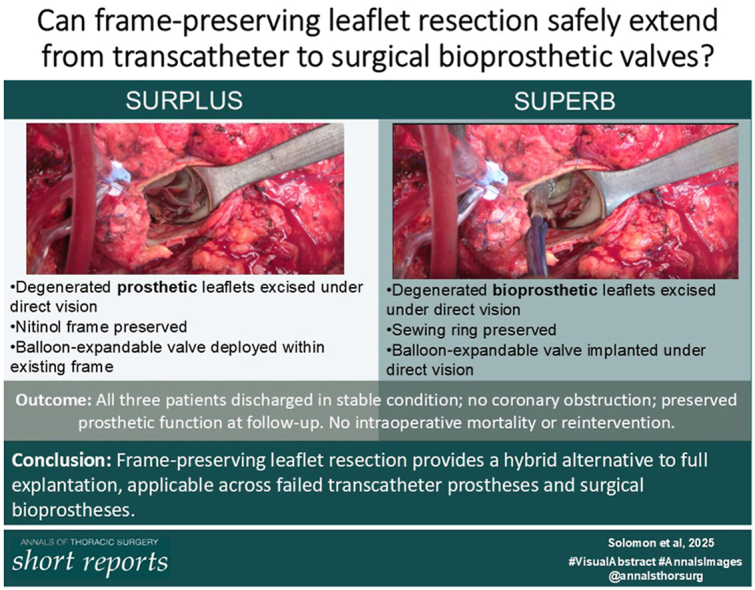

**In Short**

▪ The Surgical Valve Preservation and Resection of Bioprosthetic Leaflets (SUPERB) technique extends the Surgical Resection of Prosthetic Valve Leaflets Under Direct Vision (SURPLUS) concept to failed surgical bioprostheses.
▪ By preserving the prosthetic frame, SUPERB and SURPLUS enable safe balloon-expandable valve implantation, shorten operative times, and maintain coronary access in high-risk patients.
▪ This clinical report of SUPERB demonstrates its feasibility and highlights the complementary role of the SUPERB and SURPLUS techniques as hybrid alternatives to full prosthesis explantation.

Transcatheter aortic valve replacement (TAVR) has become a cornerstone therapy for patients with severe aortic stenosis across a wide range of surgical risk profiles.^1^ With its expanding use, structural valve degeneration, paravalvular regurgitation, and device malposition are increasingly encountered, prompting the need for reintervention^2^. Two standard strategies have evolved for that purpose: redo-TAVR (TAVR-in-TAVR), which offers a less invasive option but may be limited by anatomic constraints such as low coronary heights, pannus ingrowth, or small annuli; and surgical explantation of the prosthesis with surgical aortic valve replacement.^3^

It is worth noting that registry analyses from The Society of Thoracic Surgeons and American College of Cardiology estimate that surgical explantation after TAVR, although relatively infrequent (0.4%-1% of index cases), has one of the highest and fastest growing risk profiles in contemporary cardiac surgery, with operative mortality rates reported between 12% and 20%.^2^, ^3^, ^4^, ^5^

An alternative to the traditional surgical explantation after TAVR is described by Pirelli and colleagues^6^ as the Surgical Resection of Prosthetic Valve Leaflets Under Direct Vision (SURPLUS), which involves excising degenerated leaflets while preserving the stent frame and allows balloon-expandable prosthesis deployment under direct vision.^6^ By avoiding full explantation, SURPLUS reduces cross-clamp and bypass times, limits annular injury, and preserves coronary access.

Building on this, we introduce the Surgical Valve Preservation and Resection of Bioprosthetic Leaflets (SUPERB) technique, designed for failed surgical bioprostheses. In SUPERB, degenerated leaflets are resected while preserving the sewing ring, creating a scaffold for balloon-expandable prosthesis implantation with direct commissural alignment.

We report 3 cases: 2 using SUPERB for failed surgical valves and 1 using SURPLUS for a failed transcatheter valve, highlighting the feasibility of frame-preserving leaflet resection across valve types.

## Patients and Methods

Between January 2023 and August 2025, 3 patients with failed surgical or transcatheter bioprosthetic aortic valves underwent redo replacement at our institution (Table). All were deemed unsuitable for valve-in-valve TAVR or conventional explantation due to prohibitive risk of annular injury or coronary obstruction. Each patient was reviewed by a multidisciplinary heart team, including cardiothoracic surgery, interventional cardiology, advanced heart failure, and imaging specialists. Institutional Review Board approval was not required for this case series given the sample size of <5 patients, in accordance with institutional policy.

**Table.** Baseline Characteristics, Operative Details, Complications, and Follow-Up Outcomes of Patients Undergoing SUPERB/SURPLUS Procedure

Patient	Age (y)	Sex	Prior Valve	Comorbidities	SUPERB/ SURPLUS Procedure	Complications	Follow-Up
1	82	F	2017-Surgical bioprosthesis	CAD, AF, DM, CABG	Leaflet excision, balloon-expandable prosthesis, ascending aortic graft [SUPERB]	AKI, delirium, pneumonia	2-year echo: EF 0.60-0.65, valve normal
2	73	F	2017-Surgical bioprosthesis	HTN, AF, CVA, obesity	Leaflet excision, balloon-expandable prosthesis, ring fracture, maze + LAA clip [SUPERB]	AHRF, vasopressors, AKI	Stable, normal valve at discharge
3	83	F	2019-Transcatheter self-expanding valve	CAD, HFrEF, BiV-ICD, LBBB	Leaflet excision, balloon-expandable valve-in-frame, ICG angiography [SURPLUS]	Hypotension, urinary retention	6-mo echo: EF 0.60-0.65 valve normal

AF, atrial fibrillation; AHRF, acute hypoxemic respiratory failure; AKI, acute kidney injury; BiV-ICD, biventricular implantable cardioverter-defibrillator; CABG, coronary artery bypass grafting; CAD, coronary artery disease; CVA, cerebrovascular accident; DM, diabetes mellitus; EF, ejection fraction; F, female; HFrEF, heart failure, reduced ejection fraction; HTN, hypertension; ICG, indocyanine green; LAA, left atrial appendage; LBB, left bundle branch block; SUPERB, Surgical Valve Preservation and Resection of Bioprosthetic Leaflets; SURPLUS, Surgical Resection of Prosthetic Valve Leaflets Under Direct Vision.

Two patients with failed surgical bioprostheses underwent the SUPERB technique, defined as excision of degenerated bioprosthetic leaflets with preservation of the sewing ring, followed by deployment of a balloon-expandable prosthesis (SAPIEN S3 or SAPIEN Ultra, Edwards Lifesciences) under direct vision. One patient with a failed TAVR prosthesis underwent the SURPLUS technique, in which the prosthetic leaflets were excised but the nitinol frame was retained.

Coronary patency was confirmed intraoperatively by direct probing and, when applicable, indocyanine green (ICG) near-infrared angiography. Clinical, operative, and follow-up data were collected from institutional records and outpatient follow-up. Informed consent for publication was obtained from all patients.

## Results

### Patient 1

An 82-year-old woman with coronary artery disease, atrial fibrillation, diabetes mellitus, hypertension, hyperlipidemia, and prior coronary artery bypass grafting presented with worsening dyspnea and fatigue. She had undergone surgical bioprosthetic aortic valve replacement with a Sorin Mitroflow valve in 2017. Echocardiography revealed severe prosthetic aortic regurgitation with preserved left ventricular function.

At reoperation, dense adhesions and friable mediastinal tissue were encountered. A pulmonary artery tear during dissection was repaired with a bovine pericardial patch, and a retained pacing wire was removed. Inspection of the Mitroflow prosthesis showed severely degenerated, friable leaflets with preserved sewing ring integrity. The SUPERB technique was used to excise the leaflets and a 23-mm SAPIEN S3 valve was deployed within the preserved frame under direct vision. Coronary ostial patency was confirmed by probing. Given the aortic wall’s friability, the ascending aorta was replaced with a 28-mm Hemashield graft (Getinge) to ensure long-term durability.

The patient was weaned from cardiopulmonary bypass uneventfully. Postoperatively, she developed transient acute kidney injury requiring conservative management, delirium that resolved with supportive care, and was empirically treated for a pulmonary infiltrate. She was discharged to rehabilitation on supplemental oxygen. At the 2-year follow-up, she remained clinically stable with preserved ventricular function and a normally functioning prosthetic valve with a mean aortic gradient of 9.1 mm Hg and no regurgitation.

### Patient 2

A 73-year-old woman with multiple comorbidities, including hypertension, hyperlipidemia, atrial fibrillation, prior cerebrovascular accident, obesity, obstructive sleep apnea, and gastrointestinal bleeding, underwent Mitroflow valve implantation in 2017. She now presented with progressive dyspnea and prosthetic stenosis. Transthoracic echocardiography revealed preserved left ventricular ejection fraction (0.55-0.60), severe left atrial enlargement, and concomitant mitral stenosis and regurgitation. Computed tomography demonstrated an ascending aortic aneurysm (4.1-4.5 cm) and critically low coronary heights (right coronary artery, 4.8 mm; left main coronary artery, <5 mm), precluding valve-in-valve TAVR.

**Figure 1:**
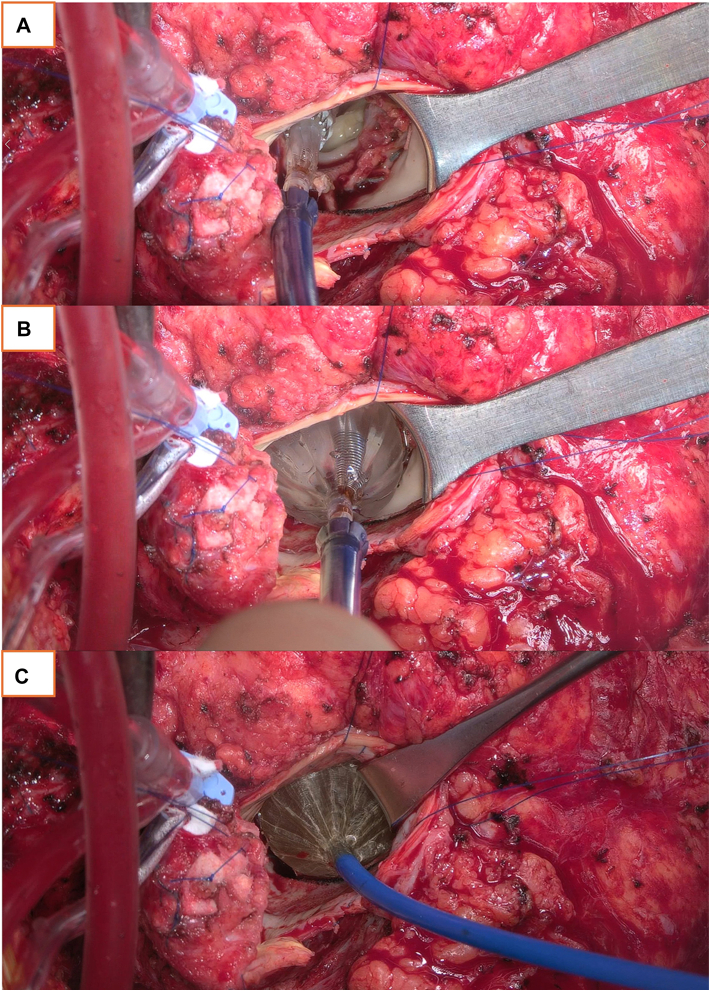
Intraoperative application of the Surgical Valve Preservation and Resection of Bioprosthetic Leaflets (SUPERB) technique with deliberate ring fracture (patient 2). (A) Excision of degenerated leaflets from a failed surgical bioprosthesis with preservation of the sewing ring. (B) Deployment of a 23-mm Edwards SAPIEN Ultra valve (Edwards Lifesciences) within the preserved ring under direct surgical vision. (C) High-pressure balloon inflation to 14 atm to deliberately fracture the surgical ring, thereby optimizing the effective orifice area and ensuring secure seating of the prosthesis.

Reoperative sternotomy was performed using an axillary artery graft and femoral venous cannulation due to severe adhesions. Concomitant procedures included modified Cox-maze ablation, left atrial appendage clipping, resection of a subaortic membrane, and complex sternal reconstruction with rib plating and bilateral pectoralis advancement flaps due to a prior Robicsek cage fracture. Intraoperatively, the Mitroflow valve was severely calcified. SUPERB was performed: degenerated leaflets were excised, the ring was preserved, and a 23-mm SAPIEN Ultra valve was implanted. To improve the effective orifice area, the prosthetic ring was deliberately fractured with a 22-mm balloon at 14 atm (Figure 1). Coronary patency was confirmed by probing.

The patient tolerated the procedure but required vasopressors for hypotension. Her recovery was complicated by acute hypoxic respiratory failure requiring ventilatory support and transient kidney injury, both resolving with supportive management. She was discharged to rehabilitation in stable condition. On follow-up, there was no valvular regurgitation, with a mean aortic gradient of 19.4 mm Hg.

### Patient 3

An 83-year-old woman with prior left anterior descending artery stent, heart failure with reduced ejection fraction (0.45), biventricular implantable cardioverter defibrillator, left bundle branch block, patent foramen ovale, hypertension, and depression presented with chest tightness and exertional dyspnea. She had undergone TAVR with a Medtronic Evolut valve in 2019. Echocardiography showed severe prosthetic stenosis, and computed tomography demonstrated circumferential pannus ingrowth into the aortic wall with very low coronary heights, rendering valve-in-valve TAVR unsafe.

**Figure 2:**
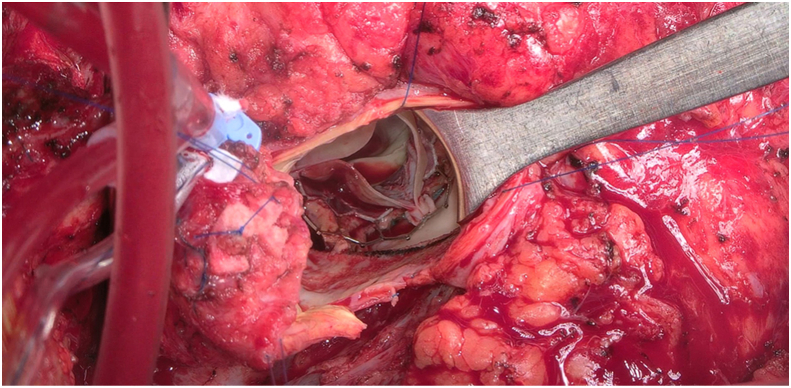
Intraoperative application of the Surgical Resection of Prosthetic Valve Leaflets Under Direct Vision (SURPLUS) technique. Degenerated valve leaflets of a failed self-expanding Evolut (Medtronic) prosthesis were excised while the nitinol frame was preserved in situ, enabling subsequent balloon-expandable valve-in-frame implantation.

**Figure 3:**
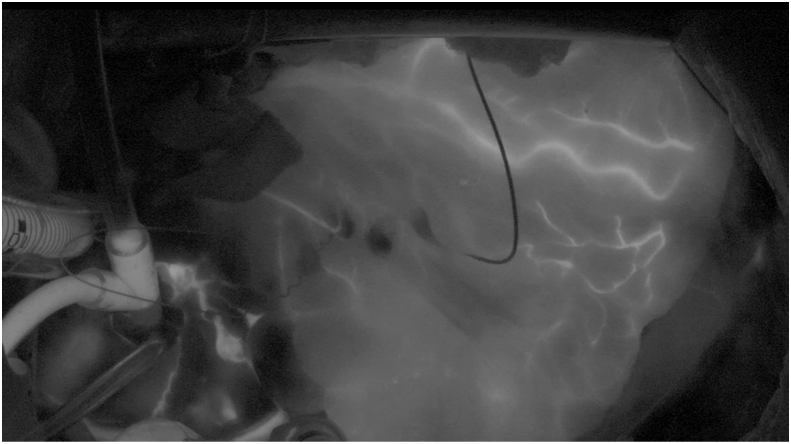
Indocyanine green infrared angiography demonstrates preserved coronary flow and ostial patency after valve-in-frame implantation with a balloon-expandable SAPIEN S3 prosthesis (Edwards Lifesciences) after Surgical Resection of Prosthetic Valve Leaflets Under Direct Vision (SURPLUS) leaflet resection.

At redo sternotomy, the Evolut prosthesis was firmly incorporated into the aortic wall, making explantation prohibitive. The SURPLUS technique was applied: degenerated leaflets were excised while the nitinol frame remained in situ. A 23-mm SAPIEN S3 valve was deployed within the Evolut frame under direct vision and secured with Ethibond (Ethicon) sutures to prevent migration (Figure 2). Coronary patency was confirmed by direct probing and by intraoperative ICG angiography (Figure 3). Intraoperative transesophageal echocardiography showed a well-seated prosthesis with preserved ventricular function.

The patient’s postoperative course was notable for transient hypotension, requiring short-term vasopressors, and urinary retention. She was mobilized early with physical therapy, tolerated diet, and was discharged to rehabilitation on postoperative day 6. At follow-up, echocardiography confirmed preserved valve function with no regurgitation and a mean aortic gradient of 22 mm Hg.

## Comment

Redo valve replacement after failed bioprostheses is a growing challenge. Although redo-TAVR is less invasive, many patients have anatomy that precludes it, and conventional explantation remains a high risk, with mortality approaching 30% in contemporary series.^3^, ^4^, ^5^^,^^7^

Frame-preserving leaflet resection strategies, such as SURPLUS and SUPERB, offer a middle ground. By excising degenerated leaflets but retaining the stent or sewing ring, operative complexity is reduced, cross-clamp and bypass times are shortened, and annular disruption is avoided. In our series, these techniques enabled safe reintervention in patients with hostile anatomy, low coronary heights, and pannus ingrowth, contexts where both redo-TAVR and full explantation posed prohibitive risk.

Direct surgical vision allows commissural alignment, promoting symmetric leaflet motion and preserving coronary access for future interventions. The ability to confirm coronary patency intraoperatively, including with ICG angiography, further enhances safety. Potential benefits include reduced conduction system trauma, lower pacemaker requirement, and more predictable hemodynamics.

Our cases illustrate complementary strengths of SUPERB and SURPLUS. SUPERB extended frame-preserving resection to failed surgical valves, demonstrating durable results at 2 years. SURPLUS enabled safe treatment of a failed TAVR with extensive pannus ingrowth without root replacement. Together, these approaches highlight a reproducible strategy applicable across valve types.

Limitations persist. These techniques are unsuitable for infective endocarditis requiring full prosthesis removal and for younger patients needing annular enlargement.^8^ Surgeons must also anticipate prosthesis-patient mismatch when implanting a balloon-expandable valve within a preserved frame, particularly in small annuli, where proximity to the sinutubular junction may impede seating. Thus, careful imaging and assessment are essential to avoid malposition. Lastly, long-term durability remains unproven.

In summary, SUPERB and SURPLUS are promising hybrid options for redo aortic valve replacement. They simplify reoperation, preserve coronary access, and avoid root reconstruction. Larger multicenter studies are needed to confirm durability and define their role in practice.
